# Extrapulmonary Nontuberculous Mycobacteria Infection: The New-Age Neglected Infectious Disease

**DOI:** 10.7759/cureus.81017

**Published:** 2025-03-22

**Authors:** Sutapa Rath, Shehnaz Firdaus, Gayatree Nayak, Monalisa Mohanty

**Affiliations:** 1 Microbiology, Yashoda Hospitals, Hyderabad, IND; 2 Microbiology, PGIMER and Capital Hospital, Bhubaneswar, IND; 3 Microbiology, Kalinga Institute of Medical Sciences (KIMS), Bhubaneswar, IND; 4 Microbiology, Dr B C Roy Multi Speciality Medical Research Centre, Kharagpur, IND

**Keywords:** diagnosis, extrapulmonary ntm infection, multidisciplinary approach, nontuberculous mycobacteria (ntm), treatment

## Abstract

Nontuberculous mycobacteria (NTM), otherwise known as atypical mycobacteria, primarily cause pulmonary disease. However, with the increase in the immunocompromised population, infections caused by NTM at extrapulmonary (EP) sites have been on the rise in the past decade. Clinical presentations can commonly include lymphadenitis and skin and soft tissue infections. The diagnosis is challenging due to the invasive nature of the sample collection and the low degree of suspicion. Furthermore, detection up to the speciation level is important as management is species-specific. With limited data and studies regarding extrapulmonary nontuberculous mycobacteria (EP-NTM) disease, a multidisciplinary approach with awareness is required to recognize the pathogen early for appropriate and timely institution of therapy.

## Introduction and background

Nontuberculous mycobacteria (NTM), also known as atypical mycobacteria or *Mycobacteria* other than *Mycobacterium tuberculosis* complex (MTBC) and *Mycobacterium leprae*, a diverse group of *Mycobacteria* representing over 200 species, are ubiquitous in the environment, including soil, dust, and natural and drinking water sources [1]. Due to their hydrophobic cell surface contributed by mycolic acid in the outer membrane, they can be acclimatized to plumbing and water distribution systems and are also resistant to different household disinfectants such as chlorine, which helps in the persistence of the organism in the water distribution system [2]. Humans get NTM infection by three common routes: inhalation, ingestion, and inoculation of the wound through injury or surgical intervention [1]. Person-to-person transmission is a very unlikely route in these infections.

The spectrum of human NTM infections largely includes four clinical syndromes: (1) pulmonary disease, especially among patients with underlying lung disease or cystic fibrosis; (2) superficial lymphadenitis, with the most commonly involved site being the cervical lymph node; (3) disseminated disease in severely immunocompromised patient conditions such as HIV, transplant recipients, and primary immunodeficiency disorders; and (4) infection involving the skin, soft tissue, bone, and joints acquired through direct inoculation [3]. The most common clinical entity is chronic pulmonary involvement, followed by lymphadenitis. Once considered an opportunistic pathogen among immunocompromised individuals, both pulmonary and extrapulmonary cases are being increasingly reported among immunocompetent individuals as well. This may be attributed to increased awareness and improved diagnostic techniques [4]. Recent record-based retrospective studies reported that extrapulmonary NTM diseases, including skin and soft tissue infections, musculoskeletal infections, lymphadenitis, and disseminated disease, contribute to 20%-30% of cases [5].

Based on their duration of growth, the species of NTM has been divided into slow growers (>7 days) and rapid growers (<7 days). The most common clinically important slow growers include *Mycobacterium avium* complex (MAC), *M. kansasii*, *M. marinum*, and *M. ulcerans*, and rapid growers include *M. abscessus* complex (MABC), *M. fortuitum* complex, and *M. chelonae* [6].

Due to their nonspecific clinical presentations, which vary widely based on the site of infection and species involved, resistance pattern to most of the commonly used antimicrobials, and lack of awareness and diagnostics facilities, the NTM disease is often misdiagnosed as drug-resistant tuberculosis, making its management further challenging [4]. This study attempts to encapsulate the grey areas such as lack of prompt diagnosis, insufficient infrastructure for rapid diagnosis, and absence of any specific treatment guidelines for extrapulmonary nontuberculous mycobacteria (EP-NTM), and try to epitomize the multidisciplinary actions needed to address the issues.

## Review

Problem statement

The rising incidence of EP-NTM infections poses a significant challenge to healthcare systems worldwide. There has been a sharp rise in global incidence with the annual prevalence increasing from 0.3 to 1 per 100,000 population [7]. Such a spike in NTM disease can be attributable to an aging population and increased use of long-term access devices and immunomodulatory therapies. These infections are often misdiagnosed or underdiagnosed due to their nonspecific clinical presentations and the lack of awareness among healthcare providers. The sample collection can be invasive, and the duration of culture is prolonged, often leading to its misdiagnosis and underdiagnosis. Moreover, the treatment of extrapulmonary NTM infections is further complicated by the intrinsic resistance of these organisms to many conventional antibiotics. This necessitates the use of prolonged and often toxic antimicrobial regimens, which can lead to adverse effects and poor patient compliance, e.g., commonly practiced treatment duration for skin and soft tissue infection is 2-4 months for mild disease and six months for severe disease, while treatment of musculoskeletal NTM disease usually requires at least 6-12 months of antimicrobial therapy [5]. Additionally, the absence of standardized diagnostic criteria and treatment guidelines further complicates the management of these infections.

Epidemiology

There has been a global concern for pulmonary and extrapulmonary infections because of their rising trend in the past decade, both in prevalence and incidence. Determining the precise burden of extrapulmonary diseases caused by NTM is difficult. Lack of knowledge regarding diagnostic modalities, their ubiquitous nature, complexities in clinical manifestations, and underreporting of NTM cases may be a few reasons for the paucity of data about these groups of infections. Systematic reporting of NTM diagnosis is not done because the disease is not notifiable to public health authorities in several countries [8].

To date, approximately 200 NTM species that most commonly cause pulmonary disease have been identified [9]. EP-NTM cases were defined by NTM isolated from any nonpulmonary body site, excluding stool and rectal swabs [10]. Although the data on extrapulmonary cases has been limited and less common compared to pulmonary ones, there has still been an increase in EP-NTM cases reported worldwide.

In a study conducted in the USA, EP-NTM had an annual prevalence of 1.4 cases per lakh population. Minnesota had the highest prevalence among all the five regions with predominance in persons aged ≥65 years. *Mycobacterium avium* complex (MAC) was isolated from 41.2% of these cases, and the sites of extrapulmonary infections were lymph nodes (19.1%), blood (17.6%), and skin (16.2%) [10]. In another retrospective analysis carried out at one of the largest academic tertiary acute care medical centers in central Florida, from January 2011 to December 2017, extrapulmonary involvement of NTM was found in 24.9% of cases. The *Mycobacterium abscessus *group was the most common, comprising *M. abscessus* subsp. *abscessus*, *M. abscessus* subsp. *massiliense*, and *M. abscessus* subsp. *bolletii*. Other rapid growers isolated were *M. fortuitum* and *M. chelonae* [11].

In a study conducted in Spain, NTM was most commonly isolated from urine, followed by abscess, skin biopsy, lymph node biopsy, and stool. NTM isolates in extrapulmonary samples were clinically significant in 100% of lymph node biopsies, 75% of skin biopsies, 41.6% of exudate/abscesses, 33.3% of stool samples, and 25.6% of urine samples, with the most common species being *M. gordonae* and *M. chelonae* [12].

In a retrospective study in a tertiary care hospital in South Korea, culture results from January 2005 to December 2019 were reviewed. This study was primarily designed to determine disseminated NTM (D-NTM) diseases in Korea. It showed that among 104 patients with extrapulmonary NTM infection, three (2.9%) were diagnosed with D-NTM disease at relatively young ages, and all had acquired or congenital immune de­fects. This study emphasized the presence of underlying immunodeficiency status in disseminated NTM disease cases [13].

Studies conducted in South Africa, Ethiopia, and Nigeria revealed a wide range of prevalence rates ranging from 0.2% to 28%. NTM infections showed a higher prevalence among HIV-infected individuals. *Mycobacterium avium* complex was reported in the majority of the cases, followed by *M. fortuitum* and* M. abscessus* [14-16].

There have been studies signifying the high prevalence of EP-NTM infections among the pediatric population. An exploratory study conducted among pediatric age groups below 14 years of age in Saudi Arabia during 2016-2017 showed 11 different species including three rare species. The slow-growing species *M. simiae* (30.8%) was predominant, followed by the fast-growing species *M. abscessus* (23.1%) and *M. fortuitum* (11.5%). These species were isolated from cases of gastrointestinal and genitourinary infection. Rare species such as *M. monacense* from ascites samples and *M. riyadhense* and *M. kubicae* from cases of lymphadenitis were also observed [17]. Similarly, to evaluate the epidemiology of extrapulmonary pediatric (children 0-14 years of age) NTM infections, a study was conducted in Queensland, Australia, where data was retrieved for the period 2000-2017. A total of 295 cases of extrapulmonary NTM infections were found, comprising 40% of skin and soft tissue infections, 33.6% cases of lymphadenitis, 12.5% from head/neck soft tissue, and 12.8% from infections present in the blood, gastric contents, feces, and unidentified body fluids [18].

In Asia, a center in Taiwan reported incidence rates of EP-NTM infections significantly rising from 1.4 to 2.23 per lakh outpatients [19]. NTM lymphadenitis was a frequent presentation, with the most commonly isolated pathogens being MAC, *M. malmoense*, and *M. scrofulaceum*. The common microbes isolated from patients with disseminated infections are MAC, *M. chelonae complex*, *M. haemophilum*, and *M. kansasii*. Skin and soft tissue infections were mostly caused by rapid growers such as *M. marinum* and *M. ulcerans* [20]. Chou et al. investigated 40 patients with disseminated NTM at a medical center in Taiwan and found that 22 (55%) of them were HIV-positive, with MAC being the most common cause in HIV-positive patients and *M. kansasii* in HIV-negative patients [21].

In Asia and Western countries, lymphadenitis is the most prevalent NTM infection in the pediatric age group irrespective of the immune status, with cervical lymph nodes being the most frequently affected site and MAC followed by rapid growers being the cause [22]. NTM prevalence has also risen among immunocompetent individuals affected with nosocomial infections. Reports from Pakistan have shown that 42% of EP-NTM infections are post-surgical, after cesarean section, laparoscopic surgeries, orthopedic surgeries with and without implants, and mastectomies. Low suspicion rates and limited access to laboratory diagnosis of NTM may likely cause this surge in infection rates [23]. Otolaryngological conditions and any history of neurosurgery or disseminated NTM might be the predisposing reasons for central nervous system (CNS) NTM disease [24]. Ocular NTM, although uncommon, has been reported in some Asian nations such as Japan and China, with a history of corneal trauma, especially caused by rapid growers such as *M. abscessus* [22].

In India, very few studies have documented the prevalence of NTM in extrapulmonary infections. In a study from North India, the prevalence of both pulmonary and extrapulmonary NTM infections was addressed; 29% of NTM were isolated, and the majority were from pulmonary cases. The extrapulmonary sites where NTM were isolated were skin and soft tissues, followed by lymph nodes and urine. *Mycobacterium abscessus* and *M. fortuitum* were the most frequently isolated NTM species from these sites [25]. A study from Hyderabad, South India, showed that the prevalence of NTM was estimated to be 3.49% among both pulmonary and extrapulmonary samples [26]. In another study from South India, NTM were isolated from 19.4% of extrapulmonary infections, and *M. intracellulare* (21.1%), *M. scrofulaceum* (15.8%), and *M. fortuitum* (10.5%) were the common isolates from them [27].

From the above epidemiological trend, it is clear that in recent years, extrapulmonary infections due to NTM have seen an upsurge. Factors responsible for this are firstly, genetic evolution in NTM due to mutation resulting in increased virulence; secondly, changes in host immunity due to chronic diseases and immunocompromised status; thirdly, increased awareness and clinical suspicion for NTM infections; fourthly, improved laboratory diagnostic tools for its identification; and lastly, due to environmental and climatic changes [28].

Risk factors

In conjunction with the virulence factors of NTM, local predisposition along with systemic immunodeficiency or certain genetic predilections can lead to nontuberculous mycobacteriosis [29,30].

Local Predisposing Factors

Any local injection or surgical or cosmetic procedure, previous history of lymphadenitis or acupuncture, trauma, any indwelling catheter, piercing, or previous history of keratitis, choroiditis, endophthalmitis, tenosynovitis, and osteomyelitis is a significant risk factor for the occurrence of EP-NTM disease. Joint replacement surgeries, vertebral disk surgery, plastic surgery, prosthetic valve replacement, and peritoneal dialysis-associated infection are surgical procedures associated with NTM infections [31-33].

Systemic Predisposing Factors

Any systemic immunodeficiency, such as HIV, malignancies receiving chemotherapy, organ transplantation, alcoholism, malnutrition, chronic granulomatous disease, use of immunosuppressive therapy, or regular use of systemic or inhaled glucocorticosteroids predisposes NTM disease. Fishery workers, fish tank owners, fishermen, seafood handlers, pet workers, and water-related recreational exposure are more common predisposing occupations. In osteoarticular infections caused by nontuberculous mycobacteria, rheumatoid arthritis, myositis, and trauma are common predispositions. Extremes of age can also predispose NTM disease [30,33].

Genetically Determined Rise in Susceptibility

Genetic mutation in the interferon-gamma (IFN-γ) receptor gene, interleukin-12 receptor gene, alpha-1 antiprotease gene, signal transducer and activator of transcription 1, and transmembrane conductance regulator gene poses an increased risk of NTM disease [34].

Genetic mutations of NTM have been leading to increased virulence. Environmental and climatic changes due to industrialization, changes in host immunity due to increased life expectancy, and a sharp increase in immunocompromised population with widespread availability of diagnostic modalities have also led to increased predisposition to disease as well as increased detection rates [28].

Pathogenesis and virulence factors

Interwoven dynamics between the host, NTM species, and environment lead to the infection. Mutations in the host's interferon-gamma (IFN-γ) pathway enhance the risk of infection due to *M. avium* and *M. abscessus*. Anti-inflammatory drugs such as tumor necrosis factor (TNF) inhibitors escalate the infections because of *M. avium* and *M. abscessus*. Additionally, broad-spectrum antimicrobials and immunosuppressants augment the chances of infection due to NTM species. Different NTM species demonstrate notable disparities in their colony morphology. Smooth and rough colony morphotypes have distinct pathological traits. The loss of glycopeptidolipid (GPL) in the rough variants promotes the conglomeration, clumping, and binding of pathogenic bacteria and the formation of biofilms, making the bacteria more resistant to hydrogen peroxide, acidic pH, sterilizing agents, and antimicrobial agents. Increased virulence has been observed in different species of NTM owing to the smooth to rough variation, e.g., *M. avium*, *M. abscessus*, and *M. kansasii*. A plethora of natural reservoirs of NTM may act as an ecological transition zone to aid in infecting the host. Contaminated water sources are the most proven environmental source of NTM infection. Showerheads are prone to NTM biofilm formation and contain more than 100-fold bacterial biomass than other household water sources [35].

Clinical features

EP-NTM infections can involve the skin and soft tissue, lymph nodes, bones and joints, urinary tract, and blood. Few reports suggest that 20%-30% of NTM infections are extrapulmonary, whereas other reports show only 4% NTM isolation from extrapulmonary organs [5,36,37]. Skin/soft tissue infections are among the most commonly affected areas, followed by blood, tenosynovium, lymph node, cornea, vertebra, gastrointestinal tract, psoas muscle, liver, and pleura, and EP-NTM has been more commonly caused by the rapidly growing atypical *Mycobacteria* [38].

Skin features may include abscesses, sporotrichoid nodules, or ulcers but can also include less distinctive signs requiring a high index of suspicion. Important species include *Mycobacterium marinum* and the rapid growers *M. fortuitum*, *M. abscessus*, and *M. chelonae* [39,40]. These microbes gain entry via skin breaks following trauma and surgical procedures, after the use of surgical instruments without appropriate sterilization, during cosmetic surgeries and pedicure and manicure procedures in beauty salons, surgical procedures involving placement of mesh materials used for hernial repairs, and various implants. Tattoo procedures using contaminated ink with *M. haemophilum* can lead to its inoculation [41], as well as intravenous punctures, intramuscular injections with contaminated needles, and usage of tap water for skin disinfection. Buruli ulcer or Bairnsdale ulcer disease, predominant in Australia and certain regions in Latin America and China, is a severe form of cutaneous infection due to *M. ulcerans* progressing from nodular cutaneous lesions into large painless ulcers, which necessitates early diagnosis and treatment to minimize morbidity and prevent long-term disability [42]. Fish tank granuloma caused by *M. marinum* can be acquired from swimming pools, cleaning of fish tanks, or any other fish- or water-related activity. Microbes generally gain access through skin cuts or abrasions. It begins as a single lesion: papulonodular, verrucous, or ulcerated granulomatous wound over the hand and forearm that proceeds to form multiple skin lesions in a sporotrichoid pattern, appearance identical to infections in cases of *Sporothrix schenckii* [43].

In the immunocompetent pediatric population, typically, cervical lymphadenitis is seen. It is often benign in nature and cured following surgical resection. The most common species responsible for lymphadenitis are *M. avium intracellulare*, *M. haemophilum*, and *M. malmoense* [32].

Iatrogenic and/or nosocomial cases are usually secondary to surgical site infections, particularly post-laparoscopic surgeries for peritoneal dialysis catheter insertion and abdominal wall abscesses following liposuctions and on prostheses [32].

In CNS nontuberculous mycobacteriosis, fever was the most predominant presenting symptom, followed by headache and altered mental state and cranial nerve involvement [44].

Disseminated EP-NTM disease is an emerging entity worldwide, particularly in immunocompromised individuals, and is characterized as an infection in two noncontiguous sterile sites or a positive result for mycobacteria from blood or bone marrow culture. Immunocompromised states include those with advanced HIV infection or long-term immunosuppression following solid organ transplantation, hematological malignancies, and chemotherapy [14].

Challenges in diagnosis and therapy

There has to be a heightened degree of suspicion as the presenting features are often similar to TB and to perform the required invasive modalities that can help the clinician acquire an appropriate sample from the site of the abscess or the affected lymph node;, granulomas, and deeper surgical sites. Even after obtaining a proper sample, the diagnosis of NTM disease is tough owing to the limited culture facilities in most healthcare centers, slow and variable growth of NTMs, and lack of molecular diagnostics. Furthermore, they can be misdiagnosed as tuberculosis in centers where only Ziehl-Neelsen (ZN) staining is used for the diagnosis of TB, and GeneXpert and culture along with *Mycobacterium tuberculosis* protein 64 (MPT64) assays are not part of the diagnostic algorithm. After appropriate speciation, treatment needs to be species-specific. However, drug susceptibility testing (DST) is not available at most facilities, and NTM species are resistant to most traditional antitubercular drugs. This further complicates the disease scenario and prolongs the time to mitigate NTM disease [32].

Evolution of diagnostic modalities over the years

The diagnosis of EP-NTM is arduous for various reasons. One reason is the variable growth patterns and divergent growth requirements of different NTM species such as rapid/slow growth rate and fastidious nature; some species require special culture media. Moreover, most pathogenic NTM species are resistant to the traditional antitubercular drugs, with variable susceptibility patterns to other classes of antimycobacterial causing non-healing infections and complications with sequelae [32]. Hence, expedited and accurate identification of NTM species is crucial for clinicians to start proper antimycobacterial agents, for patient management, and to minimize drug abuse and development of resistance.

Making an etiological diagnosis before the initiation of empirical therapy is critical as drug susceptibility tests (DSTs) vary greatly among NTM species. Mainly, three different diagnostic methods including mycobacterial culture, histopathology, and molecular methods have been used to identify NTM species. As the spectrum of the clinical manifestations due to NTM is wide, some of the cases require additional tests for diagnosis; lymphadenitis requires either excisional biopsy or fine needle aspiration cytology (FNAC), culture, and/or polymerase chain reaction (PCR). For skin infections due to NTM, skin biopsy samples are subjected to culture, histology, and/or molecular tests. In disseminated infections, mycobacterial blood, urine, and stool culture, along with bone marrow aspirate, are subjected to different tests to conclude a diagnosis [32,45,46].

Radiological diagnostic methods can be used for certain types of suspected NTM infections; for example, for a localized abscess or deep tissue infections besides bone and NTM lymphadenitis, different radiological modalities such as ultrasound, computed tomography (CT), and magnetic resonance imaging (MRI) can be utilized to identify the extent of the lesion. An MRI scan can help in cases of musculoskeletal involvement, especially when it has progressed to osteomyelitis. Involvement of the vertebra may be apparent on MRI of the spine or affected bones and joints. Positron emission tomography (PET)-CT could benefit in suspected disseminated cases. Imaging may be potentially useful to monitor treatment response, especially in inaccessible sites of abscesses where drainage is not possible [32].

Conventional Methods

Smear microscopy: Two types of stains are available for the acid-fast bacilli (AFB stain): the carbol fuchsin stain (Ziehl-Neelsen (ZN) or Kinyoun method) and the fluorochrome procedure (auramine O alone or in combination with rhodamine B) [2]. Although the specificity of ZN staining is high, its sensitivity is low (the limit of detection (LOD) is 104 bacilli/mL in the sample; sensitivity ranges from 20% to 80% depending on the quality of specimen, staining technique, microscope, expertise of the person examining the smear, and associated medical conditions such as HIV) [47]. Furthermore, AFB staining cannot distinguish between *M. tuberculosis* and NTM [45,46].

Culture: Culture remains the gold standard for laboratory diagnosis of NTM. It is a prerequisite for genotypic identification and drug susceptibility tests (DSTs). Culture media can be solid or liquid with its pros and cons. Solid media can be egg-based, such as Löwenstein-Jensen (LJ), or agar-based, such as Middlebrook 7H10 and 7H11. The advantages of solid culture media include the observation of colony morphology, growth rates, categorization of species, and quantitation of the infecting organism. Solid media can also be a backup when liquid culture media gets contaminated. An example of liquid culture media is Middlebrook 7H9 broth, the mycobacterial growth indicator tube (MGIT). The advantages of liquid media are the low limit of detection (100 bacilli/mL of sample), higher sensitivity (15% higher), and shorter turnaround time compared to solid media in detecting NTM. One disadvantage of liquid media is the higher contamination rate. So, it is recommended that all cultures for mycobacteria should include both solid and liquid media [45,46].

Biochemical tests: The common biochemical tests used for the identification include the production of niacin, nitrate reduction, tween-80 hydrolysis, urease, and catalase (qualitative and quantitative) and arylsulfatase (three days and 14 days). These tests are laborious and tedious, and sometimes, the results are inconclusive. Many of these tests have prior requirements of growth on solid media and are poorly reproducible. To date, many of the new species have not been characterized biochemically [48,49].

Despite the advancement of molecular methods, in resource-limited setups, phenotypic methods still carry an important role in the diagnosis of NTM infections.

Rapid Methods

Immunochromatographic test (ICT): Immunochromatographic test is a rapid diagnostic tool that exploits the lateral flow assay principle for detecting specific antigens or antibodies in a sample. These tests are commonly utilized in clinical microbiology due to their ease of use, rapid results, and high specificity and sensitivity. The most commonly used ICT to differentiate between *Mycobacterium tuberculosis* complex (MTBC) and NTM species is the *Mycobacterium tuberculosis* protein 64 (MPT64) antigen assay. 

Detection of MPT64 antigen: *Mycobacterium tuberculosis* protein 64 (MPT64) is an *M. tuberculosis* complex-specific antigen secreted during the growth of the bacteria. It can identify the *Mycobacterium tuberculosis* complex (MTBC) from culture material. The specificity of the MPT64 ICT kit was 100%, and its sensitivity varied between 97% and 99.1% in previous studies. Based on this principle, various commercial kits are currently available: SD Bioline, MGIT TBc Identification Test, and Capilia TB-Neo assay. It is a rapid tool used to differentiate between MTBC and NTM species [50,51].

High-performance liquid chromatography (HPLC): HPLC analyzes mycolic acids to identify species of NTM. The turnaround time is in hours. However, the high cost and need for expertise to analyze the chromatographic pattern are the major limitations of this method. The Sherlock Mycobacteria Identification System (MYCO-LS) is an FDA-cleared HPLC system for the identification of mycobacteria [52].

Molecular Methods

Nucleic acid amplification tests (NAATs): Polymerase chain reaction (PCR) is based on the amplification of specific nucleic acid sequences and offers a new diagnostic modality for the characterization of NTM with higher sensitivity and specificity. Various gene targets, such as the 16S and 23S rRNA genes, RNA polymerase beta subunit (rpoB), 65-kDa heat shock protein (hsp65), internal transcribed spacer region (ITS), and superoxide dismutase gene have been validated for mycobacterial identification. The 16S-23S ITS region varies from 270 to 360 bp between species and has greater variation in sequence than the 16S rDNA. Therefore, ITS sequencing amplifies usability in species differentiation [52]. Commercial multiplex PCR kits have been developed to identify different species of MTB and/or NTM promptly with variable sensitivity and specificity [53]. The Anyplex MTB/NTM assay demonstrated sensitivities and specificities of 1.00 and 0.96 for *Mycobacterium tuberculosis* complex (MTBC) and 1.00 and 0.97 for NTM detection in a study. Another multicenter study using Seegene Anyplex MTB/NTM MDR-TB assay for the detection of MTBC and NTM reported a sensitivity and specificity of 86.4% and 99%, respectively, for pulmonary samples and 83.3% and 100%, respectively, for extrapulmonary samples [54,55].

Line probe assay (LPA): LPA enables rapid diagnosis of NTM. In this procedure, isolation of nucleic acid (DNA or RNA) from culture or directly from the sample (respiratory samples, e.g., sputum) is done followed by amplification, and then, it is subjected to reverse hybridization onto a nitrocellulose strip with immobilized probes for different mycobacteria or for mutations that confer resistance. These strips can be quickly interpreted using a template. The turnaround time is ≤24 hours [44,56]. GenoType® Mycobacterium CM/AS (Hain Lifescience, Germany) comprises a multiplex PCR followed by reverse hybridization and line probe technology. The assay targets the 23S rRNA gene and is available in two types of kits: CM, which identifies 22 most frequently isolated species, and AS, which identifies 13 additional species. Other commercial LPA kits available are Nipro NTM+MDRTB detection kit 2 and Speed-Oligo Mycobacteria (Vircell, Granada, Spain) [47,57].

Matrix-assisted laser desorption ionization-time of flight mass spectrometry (MALDI-TOF MS): MALDI-TOF MS detects the abundance and diversity of proteins with specific mass-to-charge ratios, which is further displayed as a spectrum. The obtained spectral data are then compared to an existing database for identification. At present, two commercial MALDI-TOF MS systems are available, namely, the MALDI Biotyper (Bruker Daltonics, Billerica, MA) and the Vitek MS system (bioMérieux, Durham, NC). The accuracy of MALDI-TOF MS is dependent on both the quality of spectra obtained and the robustness of the database [5]. To date, MALDI-TOF MS has emerged as a potential tool for the accurate diagnosis of NTM species with limited availability [45].

BiDz-TB/NTM method: It is based on using BiDz sensors, which are composed of two subunits, Dza and Dzb, with target-binding fragments that are complementary to the second hypervariable region (V2) of the rrs gene encoding 16S rRNA from MTBC (Mtb-BiDz sensor), MABC, and *M. chelonae* (*M. abscessus* (Mab)/Mche-BiDz sensor), *M. avium* (Mav-BiDz sensor), *M. intracellulare*, *M. chimaera* (Mint/Mchi-BiDz sensor), and *M. kansasii* (Mkan-BiDz sensor). These BiDz sensors, in the presence of the specific target DNA, form a catalytic core that can cleave a phosphodiester bond between two ribonucleotides present in the fluorogenic substrate (MzF-FAM). The substrate is equipped with a fluorescein (FAM) fluorophore and a Black Hole Quencher at the opposite sides from the cleavage site so that the target-induced cleavage results in a fluorescent signal [58,59].

Gene sequencing is the reference method for the precise identification of NTM species. It is of great value in subspecies level identification or identification of uncommon and/or rare species. 16S rRNA gene sequencing helps to differentiate at the species level MABC. Several multi-target genes (ex-rpoB, hsp65, and ITS 16S-23S) are preferred over single-target genes for better discrimination between species [45].

Drug susceptibility test (DST): DST plays a crucial role in obtaining an ideal treatment protocol. Nevertheless, the DST for NTM is challenging due to the disparity of in vivo and in vitro results, except for amikacin and macrolides [44,60]. Slow-growing mycobacteria (SGM) associated with MAC lung disease display concordance with amikacin and macrolides; *M. kansasii* lung disease shows a positive correlation with rifampicin results. Macrolide resistance encountered in MAC lung disease is due to a mutation in the binding site of the 23S rRNA gene, mostly attributed to macrolide monotherapy. Hence, routine macrolide susceptibility testing is advised in all MAC lung disease cases, with clarithromycin being the representative of macrolides as per the Clinical and Laboratory Standards Institute (CLSI) [61,62]. Moxifloxacin and linezolid susceptibility testing should be done in macrolide resistance cases. As in most cases, treatment history is not documented, and with rifampicin being the most potent drug, rifampicin and clarithromycin were used for the primary DST for *M. kansasii*. In rifampicin-resistant isolates, the second line of antimicrobials (e.g., isoniazid, rifabutin, ethambutol, fluoroquinolones, sulfonamides, and amikacin) are tested for *M. kansasii*. In the case of rapidly growing mycobacteria (RGM), clarithromycin, amikacin, doxycycline (or minocycline), cefoxitin, ciprofloxacin, moxifloxacin, imipenem, linezolid, cotrimoxazole, and tobramycin are tested. To identify inducible macrolide resistance in RGM, particularly in *M. abscessus*, the final reading of clarithromycin should be taken after 14 days (unless resistance is detected earlier) [62].

Management of EP-NTM disease

A multidisciplinary harmonious approach is the key to the proper management of a patient with EP-NTM. This may include team effort of a range of healthcare providers such as infectious diseases, microbiology, pathology, respiratory medicine, plastic surgery or orthopedics, radiodiagnosis, and dermatology.

Treatment of EP-NTM infection

The scarcity of prospective randomized controlled trials related to EP-NTM is the key constraint for the specific treatment leading to the choice of drugs guided by pulmonary NTM infections, expert opinions, and clinical expertise. Due to discrepancies between in vivo and in vitro results, DSTs do not consistently foreshadow the clinical outcome. Additionally, the limited availability of facilities for performing DSTs and the slow growth or failure of NTM species to grow and/or degradation of drugs in cultures are other common hindrances. Whole genome sequencing may help detect mutations responsible for drug resistance, but availability is the limiting factor, especially in low-resource countries. Furthermore, treating clinicians should be cautious of the fact that, unlike TB, some species of NTM are not eradicated as per the in vitro susceptibility results. Moreover, there are no existing guidelines on the duration of treatment in the case of EP-NTM infection in contrast to tuberculosis. Treatment of EP-NTM cases is mostly guided by clinical experience, disease course, and response and should be started by a multidisciplinary team of experts, and the duration of therapy varies from six to 18 months. The treatment regime can be altered depending on the frequency and severity of adverse drug effects [32]. The examples of drugs used in some common mild and severe EP-NTM infections are depicted in Table 1, and the dosage and side effects of the drugs commonly used for the treatment are illustrated in Table 2.

**Table 1:** Treatment regimen recommended in mild and severe EP-NTM infections NTM: nontuberculous mycobacteria, EP-NTM: extrapulmonary nontuberculous mycobacteria, IV: intravenous

NTM species	Disease/infections	Recommended treatment regime for severe disease	Recommended treatment regime for mild disease
*Mycobacterium avium* complex (MAC)	Skin, soft tissue, bone, joint, tendon, and disseminated infections	Induction phase: IV amikacin + rifampicin + ethambutol + azithromycin/clarithromycin, continuation phase: rifampicin + ethambutol + azithromycin/clarithromycin	Rifampicin + ethambutol + azithromycin/clarithromycin
*Mycobacterium abscessus* complex, macrolide-resistant *M. abscessus*, macrolide-susceptible *M. abscessus*, and *M. massiliense*	Skin and soft tissue infections, stitch abscess, catheter infections, osteomyelitis peritoneal dialysis	Macrolide-resistant induction phase: IV amikacin + cefoxitin/imipenem + tigecycline + azithromycin/clarithromycin, continuation phase (3-5 of the following drugs): azithromycin, clofazimine, linezolid and quinolones, minocycline, moxifloxacin, and azithromycin	Azithromycin, clofazimine, linezolid, and quinolones
		Macrolide-susceptible induction phase: IV amikacin + cefoxitin/imipenem + tigecycline + azithromycin/clarithromycin, continuation phase: azithromycin/clarithromycin + 2-4 of the following drugs: clofazimine, linezolid and quinolones, minocycline, moxifloxacin, and azithromycin	
Mycobacterium fortuitum	Cosmetic procedures and skin and soft tissue infections in immunocompetent persons after invasive surgical procedure	Induction phase: IV amikacin + quinolone + doxycycline, linezolid can be considered, continuation phase: rifampicin + ethambutol + azithromycin/clarithromycin	Fluoroquinolone + doxycycline
Mycobacterium marinum	Localized skin infections, fish tank granuloma	Induction phase: (mostly localized lesion, so usually not required) azithromycin, clarithromycin + rifampicin + ethambutol, continuation phase: azithromycin/clarithromycin + rifampicin + ethambutol	Azithromycin/clarithromycin + rifampicin + ethambutol
Mycobacteroides chelonae	Skin infections	Induction phase: azithromycin/clarithromycin + tobramycin + imipenem, continuation phase: azithromycin/clarithromycin + clofazimine (or doxycycline, linezolid, or fluoroquinolones)	Azithromycin/clarithromycin + clofazimine or doxycycline or linezolid or fluoroquinolones
Mycobacterium ulcerans	Buruli ulcer	Induction phase: rifampicin + streptomycin, continuation phase: rifampicin + clarithromycin or moxifloxacin	Rifampicin + clarithromycin or moxifloxacin

**Table 2:** Enumeration of the dosage and side effects of the common drugs used for the treatment of EP-NTM EP-NTM: extrapulmonary nontuberculous mycobacteria, OD: once daily, IV: intravenous, ABW: actual body weight, IBW: ideal body weight, ESR: erythrocyte sedimentation rate, LFT: liver function test, SLE: systemic lupus erythematous, BM: bone marrow, ↑: increase, ↓: decrease

Name of the drug	Dosage	Side effects
Amikacin	15 mg/kg OD (max: 1 g) IV; age > 59 years: 10 mg/kg OD (max: 750 mg) IV; in obese, the dose is adjusted as per body weight	Nephrotoxicity ototoxicity and vestibular toxicity
Azithromycin	250-500 mg OD, oral	Dermatological: pruritus, rash; gastrointestinal: abdominal pain, nausea, vomiting, diarrhea, dyspepsia, alteration of taste sensation; general: fatigue; musculoskeletal and connective tissue: arthralgia; neurological: headache, dizziness, paresthesia; ophthalmic: visual impairment; ototoxicity: deafness
Rifampicin	<50 kg: 450 mg OD (oral or IV), >50 kg: 600 mg OD (oral or IV)	General: flu-like syndrome; gastrointestinal: nausea, vomiting; hematological: reversible thrombocytopenia with or without purpura (related to intermittent therapy); hepatic: transient ↑ in LFTs; neurological: headache, dizziness; other: reddish discoloration of urine, sweat, tears, and sputum; multiple drug interactions
Ethambutol	15 mg/kg OD (max: 1.2 g), obesity: where ABW > 20% above IBW	Ocular: optic neuritis; gastrointestinal: nausea, vomiting; metabolic: hyperuricemia
Clofazimine	Loading dose: 200 mg OD (oral) for 2 months, continue: 100 mg OD	Dermatological: reversible pigmentation (pink to brownish-black in 75%-100% of patients), rash, pruritus, ichthyosis; gastrointestinal: abdominal and epigastric pain, nausea, vomiting, diarrhea; ocular: ↓ in vision, conjunctival and corneal pigmentation, dryness; other: discoloration of urine, feces, sputum, and sweat; ↑ in blood sugar; ↑ in ESR
Imipenem	1,000 mg 2-3 times a day (IV), for small, frail, or elderly patients: 500 mg 2-3 times daily (IV)	Dermatological: rash; gastrointestinal: nausea, vomiting, diarrhea; hepatic: ↑ in serum transaminases, ↑ in serum alkaline phosphatase; hematological: eosinophilia
Moxifloxacin	400 mg OD, depending on weight	Gastrointestinal: nausea, vomiting, diarrhea; skin: photosensitivity, rash; hepatic: transient ↑ in LFTs; other: possible drug interactions
Doxycycline	100 mg twice daily (oral)	Dermatological: rash, photosensitivity; gastrointestinal: nausea, vomiting; neurological: headache; others: may be worsening of myasthenia gravis and SLE
Tobramycin	4.5-7 mg/kg OD (IV)	Gastrointestinal: ↓ in appetite, nausea, vomiting, diarrhea; neurological: dizziness, headache; respiratory: bronchospasm, chest discomfort, cough aphonia; nephrotoxicity, ototoxicity, vestibular toxicity
Linezolid	600 mg OD (oral or IV)	Gastrointestinal: nausea, vomiting, diarrhea; hepatic: transient ↑ in LFTs; dermatological: urticaria, rash; neurological: headache, dizziness, peripheral neuropathy; hematological: suppression of BM

Surgical intervention can be a critical component in the management of EP-NTM, and the procedures vary from debridement, excision, and drainage of the affected area. In NTM-associated bacteremia cases, removal of the device is suggested along with antimicrobial therapy for 2-3 months. Unlike TB cases, isolation is not mandatory in EP-NTM cases [32].

Due to lack of any proper treatment guidelines for EP-NTM disease, the panel of experts mostly rely on the guidelines provided by the joint effort of the American Thoracic Society (ATS), European Respiratory Society (ERS), European Society of Clinical Microbiology and Infectious Diseases (ESCMID), and Infectious Diseases Society of America (IDSA) for the treatment of nontuberculous mycobacterial (NTM) pulmonary disease in adults (2020) [63]. Treatment for EP-NTM infections must only commence by a team of experts from multiple dimensions of healthcare with experience in the management of patients with EP-NTM.

Due to the lack of any proper treatment guidelines for EP-NTM, the majority of drugs used off-license in the treatment of NTM in the UK. Depending on the renal and liver function, the dosage of the drugs is adjusted to avoid side effects [32].

Way forward

To effectively address the growing burden of extrapulmonary nontuberculous mycobacteria (EP-NTM) infections, an all-inclusive approach is the need of the hour. The authors propose a multidimensional approach that includes enhanced diagnostic methods, standardized treatment protocols, and increased awareness among healthcare providers. New-age researchers should prioritize their research on understanding the regional epidemiology of EP-NTM infections, which will effectively fine-tune public health measures. Developing rapid, accessible, and affordable diagnostic tools is essential for improving early detection and differentiating these infections from other mycobacterial diseases, especially drug-resistant tuberculosis. Establishing a clinical evidence and susceptibility pattern-based treatment protocol will also support better managing EP-NTM infections. Moreover, a collaborative effort among microbiologists, clinicians, public health experts, and policymakers is crucial to alleviate the EP-NTM infection menace. Finally, prioritizing prevention strategies, including strong infection control measures and public health education including strengthening the surveillance at the primary healthcare level and improving the referral facilities, will be fundamental in controlling the spread of these infections.

## Conclusions

Extrapulmonary NTM infections have emerged as a notorious yet often overlooked public health issue over the years. Its prevalence is upsurging in parallel with the increasing cases of immunosuppressants, improved diagnostic modalities, and better medical interventions. Still, unlike tuberculosis, EP-NTM infections lack standardized global treatment guidelines, resulting in delays in diagnosis and inconsistent management. Hence, the growing burden of EP-NTM infections necessitates better awareness among healthcare providers, the development of diagnostic algorithms, the amelioration of laboratories, and the establishment of resistance detection methods to ensure effective tailored therapy to curb their effects on vulnerable populations.
